# Annotations

**Published:** 1890-04-26

**Authors:** 


### Annotations.

**The College of Physicians.**

Sir Andrew Clark has been elected for the third time President of the Royal College of Physicians of London. That body has shown a just appreciation of professional and general merit in its choice. Sir Andrew Clark has not always succeeded in satisfying everybody, or in commending his line of action to universal approval. But who has performed a feat of such an extraordinary kind as that? Certainly no President of the College of Physicians. We are glad that Sir Andrew remains at the head of affairs in the medical profession. So long as he is there it is certain that conscience, capacity, culture, diligence, and experience will be available for the business of the College. Nobody who knows Sir Andrew Clark, however much they may at times differ from his judgments, doubts the goodness of his heart or the admirable qualities of his brain. The College greatly needs the illumination that superior intellects can bestow upon it. An inheritance of narrow, illiberal, and half-cultured traditions cannot be got rid of in a decade. We sympathise profoundly with the conservation of all that is truly venerable. Traditions that are merely harmless, although they may sometimes seem to border on the ridiculous, need not be recklessly cast aside. But when tradition fetters the freedom of the intellect, when it shuts out the light of a true and searching science, when it hides its own ignorance from the general gaze in clouds of mystery and fogs of unworthy expediency, when, in short, it makes a scientific and honest profession "suspect" to the candid mind of the great majority of men, then it is to be regarded as a common enemy, and as a common enemy it is to be exposed and denounced. The College of Physicians is happily beginning to recognise that daylight and sunshine are to be welcomed everywhere. The foundations of religion have been searched and exposed by the critical eye, with the result that every free and honest soul is made glad, and holds his religion with tenfold more reverence and affection than before. Let the foundations of medicine be laid bare by the same stern means. The men of the past are dead, and they, therefore, can feel no shame. The men of the present have nothing to be ashamed of, but the contrary. The physiology and medicine of the last few decades have not only been eminently honest and scientific in themselves, but have set before all the world an example of unflinching loyalty to everything of the nature of fact. That example has had a potent influence on religion, politics, business, and every other department of life. Under Sir Andrew Clark we may reasonably hope that the College of Physicians will more and more sever itself from that which is unworthy of its high position, and commit itself loyally and unreservedly to the honest and open daylight of modern and scientific ways.

**The late Bishop Callaway.**

The Right Reverend Henry Callaway, late Bishop of Kaffraria, was M.D. and M.R.C.P., as well as D.D. When a Doctor of Medicine becomes a teacher of religion, there is a feeling that a happy combination of callings has been made. It is a little surprising that young medical men do not offer themselves in larger numbers as propagandists of the Christian religion. The explanation is probably to be found in the rigid, mechanical, and unnatural forms into which Christianity has been forced by the various churches. Bishop Callaway, as the story of his life shows, was always a deeply religious man; but he seems to have spent thirteen or fourteen of the most active years of his life in settling the question as to what church most truly represented Christianity. That we venture to think is a reproach to all the churches. It is probable that many deeply earnest and practically able men are kept out of ministerial and missionary service by exactly such difficulties as hindered Dr. Callaway. We ourselves know some such men. Dr. Callaway seems never to have become a typical ecclesiastic. He always remained a man and a physician as well as a shepherd of souls. It does not appear that he was a worse bishop on that account; on the contrary, he was manifestly a better one. Mr. Jonathan Hutchinson furnishes one or two retrospective stories to the *British Medical Journal*, which show excellently well what kind of sincere, conscientious, and brotherly soul the late Bishop always was. Bred in the Church of England, he became for a time a member of the Society of Friends, apparently as the result of associating with the late Mr. May in the capacity of assistant. With the zeal of a convert, he soon outran Mr. May in the direction of stern Puritanism. Said Mr. May to Mr. Hutchinson, on one occasion, "Henry Callaway has been reproving me for allowing *Punch* to come into my family; dost thou think there is any harm in it?" This is delicious! Mr. Hutchinson tells another story which as charmingly illustrates Callaway's conscientious simplicity of character. When a student at St. Bartholomew's, he, on one occasion, thought it his duty to offer a rebuke to one of his teachers, the late Mr. Wormald, and thus addressed his reverend senior: "I am grateful, Thomas Wormald, for much practical knowledge which thou hast given me; but thou must permit me to say that I have often been pained by the anecdotes thou relates. Excuse me saying it, but thou art too jocose." Many a five hundred paged memoir has failed to give such a clear insight into the character of its subject as is conveyed by these two little stories. No doubt a life of Dr. Callaway will soon be forthcoming, and if the writer does anything like justice to his subject the book cannot fail to exercise an excellent influence upon both the clergy and the medical profession. As we so constantly reiterate here, men should be men first and specialists afterwards. Dr. Callaway was always pre-eminently a man.

**The Indoor Staff at Stockton.**

The late Mr. W. E. Forster, M.P., once reminded an Edinburgh audience of a Yorkshire saying—"If you scratch a Yorkshireman you find a Scotchman." The men of Yorkshire and those north of the Tweed have this in common, that they like to get full value for their money. A case in point is the work on the one hand, and the salary on the other, of the House Surgeon of the Stockton-on-Tees Hospital. That official has hitherto had under his sole charge a hospital of twenty-four beds, and in addition has had to give two hours in the morning and two in the afternoon every day to the work of the dispensary, an institution altogether distinct and separate from the hospital. For all this he has received a salary of £200 a year. The pay, it must be admittted, is fairly liberal, but the work has certainly been excessive, if it has been at all well done. Now, however, a new set of circumstances is emerging. The hospital is more than doubled in size, and will shortly have fifty in-patients. The problem which is vexing the souls of the managers is whether the house surgeon will be able to attend properly to fifty in-patients, and to still give four hours a day to the dispensary. The chairman of the committee is apparently of opinion that there is no difficulty at all in the matter. If he is correctly reported, he thinks that past generations of house surgeons at Stockton would have had nothing to do but "idle about half their time," if it had not been for the saving necessity of attending to the Dispensary. We appreciate the keenness of this Yorkshireman's teeth; but we are emphatically of opinion that if the House Surgeon had attended to his twenty-four in-patients as modern scientific medicine insists that they ought to be attended to; and if he had kept his case-books and other necessary papers as they are now required to be kept, he would have had no time to spare for any of the work of the Dispensary. To suppose that when there are fifty patients instead of twenty-four at the Hospital, the House Surgeon will have any time at all for the Dispensary is ridiculous. The true solution of the difficulty is this: let some junior practitioner in the town be appointed to the Dispensary; and as for the Hospital, let two house surgeons, or a house physician and a house surgeon be appointed, the senior with £100 and the junior with £50 a-year. If this be done the Hospital will be able to command the services of able and scientific young men; their methods of work will react favourably on the honorary staff; and thus not only will the Hospital patients be better treated, but the whole tone of medical practice in Stockton will be raised. Economy is generally sound policy, but cheese-paring is a very unprofitable occupation—a fact which nobody ought to know better than a Yorkshireman.

